# The Dental Department of the University of Maryland

**Published:** 1882-05

**Authors:** 


### EDITORIAL, ETC.

*The Dental Department of the University of Maryland.*—During the last session of the Maryland State Legislature a charter for a Dental Department was granted in connection with the School of Medicine and Law, the University School of Medicine being the third oldest institution of the kind in this country. The newDental Department has been organized by the appointment of the following faculty:

Ferdinand J. S. Gorgas, M. D., D. D. S., Professor of Principles of Dental Science, Dental Surgery and Mechanism. James H. Harris, M. D., D. D. S., Professor of Operative and Clinical Dentistry. Wm. E. A. Aikin, M. D., L. L. D., Professor of Chemistry. Samuel C. Chew, M. D., Professor of Materia Medica and Therapeutics. Francis T. Miles, M. D., Professor of Physiology. L. McLane Tiffany, M. D., Clinical Professor of Oral Surgery. J. Edwin Michael, M. D., Professor of Anatomy. John C. Uhler, M. D., D. D. S., Demonstrator of Mechanical Dentistry. Frank L. Harris, aud Lewis M. Cowardin, D. D. S., Demonstrators of Operative Dentistry. Charles L. Steel, M. D., D. D. S., B. M. Hopkinson, D. D. S., Assistant Demonstrators of Operative Dentistry, and Thomas H. Parramore, D. D. S., Howard W. Hoopes, D. D. S., Charles F. Dinger, D. D. S., and Luke J. Pearce, D. D. S. Assistant Demonstrators of Mechanical Dentistry. Randolph Winslow, M. D., Demonstrator of Anatomy. Alsoa large corps of eminent and experienced Clinical Instructors.

Prof. F. J. S. Gorgas has been connected with the old Baltimore College as Professor and Dean for a period of over *twenty-five* years, and during that time has occupied every chair and demonstratorship, except that of Chemistry. Prof. James H. Harris has occupied the Chair of Clinical Dentistry in the old Baltimore College for eleven years. Dr. John C. Uhler, the Demonstrator of Mechanical Dentistry in the new School, has occupied the same position in the old Baltimore College for eight years. Prof. Ferdinand J. S. Gorgas is the Dean of the Dental Department of the University, his address being the same as for many years past, 259 N. Eutaw Street.

A new building is now being erected for the Dental Infirmary and Laboratory which will not only be one of the largest, but the best lighted and equipped in the world. There will be in the Infirmary twenty-five large windows on *every* side, and its position running East and West, will make it one of the most comfortable as well as the most convenient of structures for such purposes. The Infirmary will extend over the entire second story and the Laboratory will occupy the first story, and both be equipped with entirely new and the most recent appliances. This new building will be completed and occupied by the first of August next.

The large building known as Practice Hall will be used as a Dental Lecture Hall, above which is the large and interesting Museum of the University.

The Summer Session commenced on the 15th of the present month, May, and is held in the large Dispensary Hall of the University Hospital Building Twenty-four thousand patients visited this large Hospital during the past year, which institution will furnish all the dental material desired for Infirmary Patients. Students attending the Summer Session have the privilege of attending the daily Surgical and Medical Clinics of the University.

Besides this Hospital, there are a number of Charitable Institutions in charge of the Faculty of the University, which will also furnish a large number of patients for the Students of the dental department. The lectures on Oral Surgery and the Oral Surgery Clinics will prove of inestimable value to the dental students, who have the privilege of attending all the general surgical clinics of the different Professors of the Medical Department. The instruction in both operative and mechanical dentistry will be as thorough as is possible, and the Dental Laboratory will be so equipped and arranged as to make it superior to any private laboratory.

The regular session will begin October 2nd, 1882, and continue until the latter part of February or 1st, of March, ensuing.

The fee sand terms are the same as at other Dental Colleges with the advantage of graduation in medicine at the end of one year after graduation in dentistry. Beneficiary Students are received from every State Dental Society at half fees, and no fees are charged for the Summer Session.

